# Flagellar Synchronization Is a Simple Alternative to Cell Cycle Synchronization for Ciliary and Flagellar Studies

**DOI:** 10.1128/mSphere.00003-17

**Published:** 2017-03-08

**Authors:** Soumita Dutta, Prachee Avasthi

**Affiliations:** a Department of Anatomy and Cell Biology, University of Kansas Medical Center, Kansas City, Kansas, USA; b Department of Ophthalmology, University of Kansas Medical Center, Kansas City, Kansas, USA; University at Buffalo

**Keywords:** *Chlamydomonas*, flagellar length, precursor pool, regeneration, synchronization

## Abstract

The unicellular green alga *Chlamydomonas reinhardtii* is an ideal model organism for studies of ciliary function and assembly. In assays for biological and biochemical effects of various factors on flagellar structure and function, synchronous culture is advantageous for minimizing variability. Here, we have characterized a method in which 100% synchronization is achieved with respect to flagellar length but not with respect to the cell cycle. The method requires inducing flagellar regeneration by amputation of the entire cell population and limiting regeneration time. This results in a maximally homogeneous distribution of flagellar lengths at 3 h postamputation. We found that time-limiting new protein synthesis during flagellar synchronization limits variability in the unassembled pool of limiting flagellar protein and variability in flagellar length without affecting the range of cell volumes. We also found that long- and short-flagella mutants that regenerate normally require longer and shorter synchronization times, respectively. By minimizing flagellar length variability using a simple method requiring only hours and no changes in media, flagellar synchronization facilitates the detection of small changes in flagellar length resulting from both chemical and genetic perturbations in *Chlamydomonas*. This method increases our ability to probe the basic biology of ciliary size regulation and related disease etiologies.

**IMPORTANCE** Cilia and flagella are highly conserved antenna-like organelles that found in nearly all mammalian cell types. They perform sensory and motile functions contributing to numerous physiological and developmental processes. Defects in their assembly and function are implicated in a wide range of human diseases ranging from retinal degeneration to cancer. *Chlamydomonas reinhardtii* is an algal model system for studying mammalian cilium formation and function. Here, we report a simple synchronization method that allows detection of small changes in ciliary length by minimizing variability in the population. We find that this method alters the key relationship between cell size and the amount of protein accumulated for flagellar growth. This provides a rapid alternative to traditional methods of cell synchronization for uncovering novel regulators of cilia.

## Abstract (precis)

Cilia and flagella are highly conserved antenna-like organelles that found in nearly all mammalian cell types. They perform sensory and motile functions contributing to numerous physiological and developmental processes. Defects in their assembly and function are implicated in a wide range of human diseases ranging from retinal degeneration to cancer. *Chlamydomonas reinhardtii* is an algal model system for studying mammalian cilium formation and function. Here, we report a simple synchronization method that allows detection of small changes in ciliary length by minimizing variability in the population. We find that this method alters the key relationship between cell size and the amount of protein accumulated for flagellar growth. This provides a rapid alternative to traditional methods of cell synchronization for uncovering novel regulators of cilia.

## INTRODUCTION

The unicellular, biflagellate alga *Chlamydomonas reinhardtii* is extensively used as a model organism for studying fundamental processes such as photosynthesis, cell motility, cell signaling, cell-cell recognition, and regulation of ciliary assembly-disassembly (1). This organism offers many advantages for molecular and biochemical studies of eukaryotic flagella, as their flagellar structure and function are well conserved (2). *Chlamydomonas* cells can be chemically or mechanically induced to shed their flagella (termed “deflagellation”). After amputation, they can regenerate flagella to predeflagellation lengths rapidly (within 2 h). Flagellar assembly and disassembly are precisely controlled throughout cell cycle progression and cell division (3, 4). During cell division, flagella are disassembled naturally. Flagellar resorption starts at the preprophase stage and continues about 30 min prior to mitotic cell division (5). New flagella begin to form in the daughter cell after division (6, 7). During the sexual cycle, flagella begin to resorb a few hours after the fusion of gametes, and the process proceeds gradually as in vegetative growth (8).

As cell division plays a critical role in flagellar growth and resorption, cultures with a heterogeneous population of cells in different divisional stages differ in flagellar length (F-L). In contrast, synchronous cultures, which contain cells that are in the same growth stage, have a comparatively homogeneous distribution of flagellar lengths. Thus, synchronous cultures provide advantages over nonsynchronous cells for studying cellular morphology and the effects of various chemical or genetic perturbations on flagellar length.

A wide range of physical and chemical methods have been applied to achieve synchronization for different cells or tissue types. Synchronization of bacteria can be carried out by single or multiple changes of temperature or light, single or multiple cycles of nutritional starvation, cell cycle inhibitor block, and size selection by filtration or centrifugation (9–12). Fission yeast can be synchronized either by separating a subpopulation from an asynchronous culture using specialized centrifugation or by selecting cells from a lactose gradient (13). Temperature-sensitive cell cycle mutations or inhibitors are also used to block the cell cycle at different stages of growth, allowing cells to grow synchronously upon withdrawal of the block (14). Common methods for mammalian cell cycle synchronization are inhibition of DNA replication (15) and inhibition of mitotic spindle formation using different chemical inhibitors (16–18). Nonchemical methods for cell cycle synchronization include amino acid and serum starvation (19). Cells can also be mechanically separated by physical methods such as flow cytometry, mitotic shake-off, and countercurrent centrifugal elutriation (18). Hypoxic shock and hyperthermic shock have been used to synchronize cells of the ciliate *Tetrahymena pyriformis* (20). Photosynthetic algal cells are typically exposed to alternative light/dark (L-D) cycles for synchronization (21, 22).

In *Chlamydomonas reinhardtii*, as in other photoautotrophic cells, the most common method used for cell synchronization is alternating light/dark cycles (12 h/12 h or 14 h/10 h) in minimal medium (23, 24), though other conditions and other methods such as periodic hypothermic conditions (25), selection by size (26), and variable wavelengths of light (27) have been applied. Synchronization can also be achieved by incubating *Chlamydomonas reinhardtii* cells in low-nitrogen medium for at least 15 h (28). *Chlamydomonas* cells undergo gametogenesis upon nitrogen deprivation (using nitrogen-free minimal medium [M-N]). After induced gametogenesis, culture contains mostly new-born cells with smaller sizes (29–31). During L-D synchronization, cells can grow during the light phase to many times their original size (32). In the dark phase, cells can undergo consecutive divisions to produce 2, 4, 8, 16, or even 32 daughter cells depending on the cell size (33). Cells divide in the middle of the dark cycle in *Chlamydomonas reinhardtii* (23), whereas the division occurs in *Chlamydomonas moewusii* cells during the late phase of darkness (34). Although cell division is restricted to each dark phase, the starting times of individual cell divisions differ from cell to cell. Thus, consecutive cell divisions take place throughout several hours of the dark period. As a result, the cells are always partially asynchronous in their division at any point in time (8). In addition, cultures are maximally synchronized only after the third iteration of light-dark cycling since some populations of the cells divide during the first and second iterations of the light phase (23). Different factors such as light duration and intensity, temperature, and culture density also have an effect on the degree of homogeneity (32, 35). For example, in *Chlamydomonas eugametos*, L-D synchronization can be achieved only if the culture is static without aeration (36).

While synchronization of mammalian cells can be optimized, it is not possible to synchronize entire cell populations by any of the methods or techniques described above (37). Traditional L-D synchronization or nitrogen starvation methods can only make partially synchronized *Chlamydomonas* cultures. As cells are not truly synchronized using these methods, high variabilities of flagellar length are still observed within the population. If the culture contains too much heterogeneity, it can be difficult to detect effects of flagellar length perturbations. As synchronized cells are ideal for assaying length-related flagellar dynamics, here we have outlined and characterized a method in which 100% of cells are synchronized with respect to their flagellar length but are not synchronized with respect to the cell cycle. We tested the utility of this method in evaluating flagellar length after chemical and genetic perturbations. Finally, we probed the basis of flagellar length synchronization by probing the synthesized but unassembled pool of flagellar protein.

## RESULTS

### Flagellar length synchronization narrows the steady-state flagellar length distribution.

**FIG 1:**
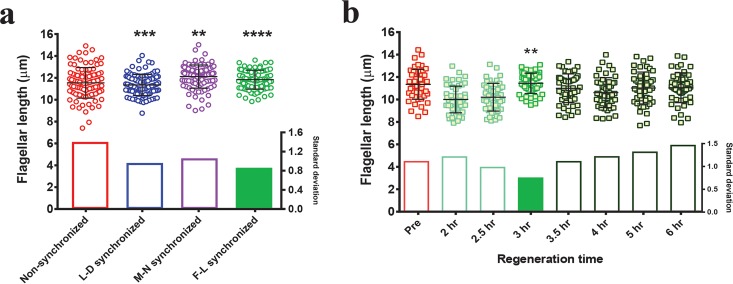
F-L synchronization narrows the flagellar length distribution compared to other synchronization methods. (a) Distribution of steady-state flagellar lengths after the use of different synchronization methods. Nonsynchronous cells were used as a control, and steady-state flagellar lengths were measured after the use of each synchronization method as described in the text (*n =* 100/each). The *F* test was performed for comparing variance levels, and two-tailed *P* values were determined. Asterisks indicate significant differences (****, *P* ≤ 0.0001; ***, *P* ≤ 0.001; **, *P* ≤ 0.01). Standard deviations of each distribution are shown below the individually plotted values. (b) Wild-type flagellar length distribution at various time intervals during the regeneration after amputation. Predeflagellation nonsynchronous cells (pre) are shown in red. Regeneration was carried out for the indicated times after deflagellation by pH shock (green). Lighter green and darker green indicate before and after the time of F-L synchronization, respectively (*n =* 50/each). The *F* test was performed for comparing variance levels (control = predeflagellation nonsynchronous cells). Bonferroni corrected α_altered_ = 0.007. The asterisks indicate significant difference below α_altered_ (**, *P* = 0.004). Standard deviations are expressed as bar graphs in the bottom half of the panels. The filled standard deviation bar represents the condition with the smallest variance.

Synchronous culture provides a better way to address cell cycle and related flagellar dynamics. However, cell cycle synchronization methods provide only partial synchronization and thus show high variance in flagellar length. To obtain 100% flagellar length (F-L) synchronization, we exploited an inherent property of *Chlamydomonas*, which is their ability to regenerate the flagella after amputation (38). We first performed different synchronization methods and then compared their steady-state flagellar lengths to those of nonsynchronized cells (Fig. 1a, Fig. S1 in the supplemental material, and Table S1 in the supplemental material). For F-L synchronization, we tested different regeneration time durations following deflagellation to determine the time point at which flagellar length variability is minimized. We found that the predeflagellation flagellar length distribution was broad, which was expected as our starting culture was nonsynchronous and contained a heterogeneous population of cells (Fig. 1b, red). After deflagellation, all flagella started to grow synchronously but the length distribution still remained broad at 2 and 2.5 h, when some cells were still in the 8- to 9-µm size range and did not reach their original length (Fig. 1b, light green). The distribution narrowed and became maximally homogeneous at 3 h (Fig. 1b, medium green) (Table S2). However, the length distribution remained narrow for only a short time, expanding again within 30 min and increasing with time (Fig. 1b, dark green). This timing was highly reproducible, and the data shown in Fig. S2 represent the combined results of three independent experiments. On the basis of the standard deviations (SD) of these distributions (Fig. 1b, lower panel) (Fig. S2, lower panel), we selected a regeneration time of 3 h postdeflagellation as the flagellar length synchronization time and the time at which to initiate further experiments. Likewise, after determining conditions of minimal variability for each synchronization method, we measured the steady-state flagellar lengths for comparative analysis. The results revealed that the mean flagellar lengths at steady state were almost equivalent for all methods (Table S1). As expected, nonsynchronous cells had larger variability than all synchronized cells (Fig. 1a, red). The F-L synchronization method shows a remarkably narrow spread of measurements around the mean and the lowest variability of length across all synchronization methods (Fig. 1a, green, and standard deviation bar graph below) (Table S1). In contrast, conventional L-D synchronization and M-N synchronization have comparatively wider distributions (Fig. 1a, blue and purple, respectively) (Table S1). Our findings suggest that the F-L synchronization is the most effective method for achieving maximum flagellar length homogeneity.

10.1128/mSphere.00003-17.1 FIG S1  Distribution of steady-state flagellar lengths after the use of different synchronization methods. F-L synchronization narrows the flagellar length distribution compared to other synchronization methods. Combined data from three independent experiments are represented. *n =* 100/each; total, 300. Nonsynchronous cells were used as a control, and steady-state flagellar lengths were measured after employment of each synchronization method as described in the text. The *F* test was performed for comparing levels of variance, and two-tailed *P* values were determined. Asterisks indicate significant differences (****, *P* ≤ 0.0001; *, *P* ≤ 0.05). Standard deviations of each distribution are shown below the individually plotted values. Download FIG S1, PDF file, 0.1 MB. Copyright © 2017 Dutta and Avasthi. 2017 Dutta and Avasthi This content is distributed under the terms of the Creative Commons Attribution 4.0 International license.

10.1128/mSphere.00003-17.6 TABLE S1  Flagellar length distribution after the use of different synchronization methods. Download TABLE S1, PDF file, 0.03 MB. Copyright © 2017 Dutta and Avasthi. 2017 Dutta and Avasthi This content is distributed under the terms of the Creative Commons Attribution 4.0 International license.

10.1128/mSphere.00003-17.2 FIG S2  Wild-type flagellar length distribution at various time intervals during the regeneration after amputation. Predeflagellation nonsynchronous cells (pre) are shown in red. Regeneration was carried out for the indicated times after deflagellation by pH shock (green). Lighter green and darker green indicate the times before and after F-L synchronization, respectively. Combined data from three independent experiments are represented (*n =* 50/each; total, 150). The *F* test was performed for comparing levels of variance (control = nonsynchronous cells). Bonferroni corrected α_altered_ = 0.007. The asterisks indicate significant difference below α_altered_ (***, *P* = 0.0006). Standard deviations are expressed as bar graphs in the lower panel. The filled standard deviation bar represents F-L synchronization. Download FIG S2, PDF file, 0.1 MB. Copyright © 2017 Dutta and Avasthi. 2017 Dutta and Avasthi This content is distributed under the terms of the Creative Commons Attribution 4.0 International license.

10.1128/mSphere.00003-17.7 TABLE S2  Distribution of flagellar lengths during regeneration following deflagellation. Download TABLE S2, PDF file, 0.03 MB. Copyright © 2017 Dutta and Avasthi. 2017 Dutta and Avasthi This content is distributed under the terms of the Creative Commons Attribution 4.0 International license.

### Increased effects of chemical perturbations using F-L synchronization.

**FIG 2:**
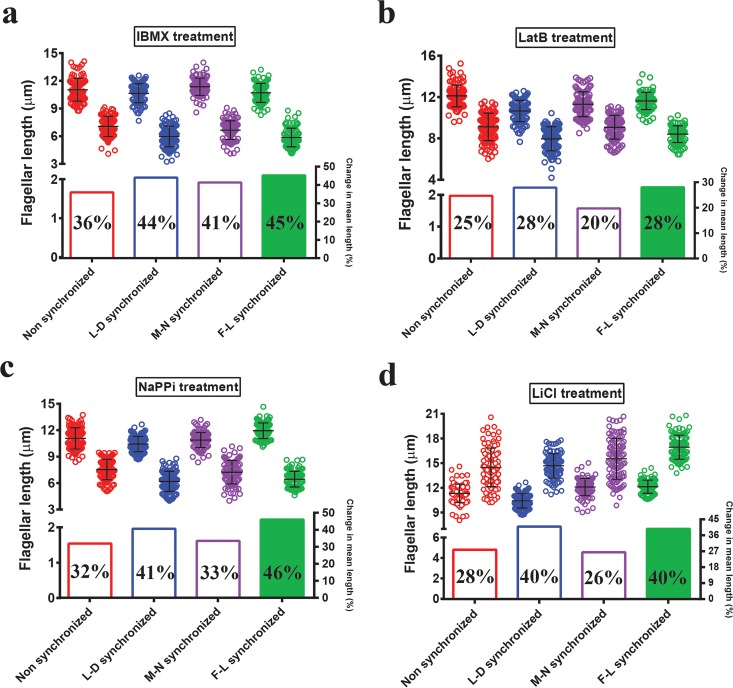
Effects of length-altering chemical treatments are more apparent after F-L synchronization. Flagella were measured from each cell after incubation with the appropriate concentration of different chemicals for 90 min. (a) 0.4 mM IBMX. (b) 10 µM LatB. (c) 10 mM NaPPi. (d) 25 mM LiCl. For each pair: red, nonsynchronized; blue, L-D synchronized; purple, M-N synchronized; green, F-L synchronized. The first and second distributions for each pair represent control and treated conditions, respectively. *n =* 100 cells (one flagellum per cell). Bars represent means and standard deviations (top half of each panel). In each case, percent change in mean flagellar length is shown below individual plotted values.

Flagellar length can be perturbed chemically. If the perturbation has a small effect on flagellar length, high variance in the system may mask observed phenotypes. Our observations demonstrate that F-L synchronized cells have reduced variance in flagellar length compared to nonsynchronized cells synchronized using other methods (Fig. 1a) (Fig. S1). Therefore, we tested the effects of several known flagellar length-altering agents after reducing variability in the initial population through F-L synchronization and compared the results with those obtained by other synchronization methods. When flagellar shortening was induced with 3-isobutyl-1-methylxanthine (IBMX) (39), latrunculin B (LatB) (40), and sodium pyrophosphate (NaPPi) (41), we observed more severe shortening in F-L synchronized cells than in nonsynchronized cells or cells synchronized using all the other methods (Fig. 2a to c, green) (Table S3). Only L-D synchronization, which is a more time-consuming synchronization method, demonstrated length reduction comparable to that seen with F-L synchronized cells (Fig. 2a to c, blue) (Table S3). Effects on flagellar length were the most extreme in the case of NaPPi-mediated length resorption (Fig. 2c). After the treatment, flagellar length distribution was significantly reduced in F-L synchronized cells compared to the others and produced a more dramatic shortening of the mean flagellar length (Fig. 2c) (Table S3). L-D synchronized cells showed a reduced effect (~41% shortening) compared to F-L synchronized cells (~46% shortening) (Fig. 2c, lower panel). In addition to testing flagellum-shortening compounds, we also tested the effects of lithium chloride (LiCl), which is known to lengthen flagella (6). The flagella were longest in F-L synchronized cells after LiCl treatment. While the levels of variance and percent change in mean flagellar length were comparable between L-D and F-L synchronized cells, each of the LiCl-treated F-L synchronized cells had flagellar length greater than 13.5 µm, with an average of 17 µm (Fig. 2d, green) (Table S3). Broad distributions of lengths ranging from 9 µm to 20 µm were observed in both nonsynchronized and M-N synchronized cells (Fig. 2d, red and purple, respectively). As a result, the effect on flagellar length was less apparent. Taken together, all of these data demonstrate that the effect of each chemical is more prominent and detectable in F-L synchronized cells when the variance in starting flagellar length is minimized.

10.1128/mSphere.00003-17.8 TABLE S3  Flagellar length distribution after length-altering chemical treatment. Download TABLE S3, PDF file, 0.04 MB. Copyright © 2017 Dutta and Avasthi. 2017 Dutta and Avasthi This content is distributed under the terms of the Creative Commons Attribution 4.0 International license.

### Synchronization time varies in flagellar length mutants.

**FIG 3:**
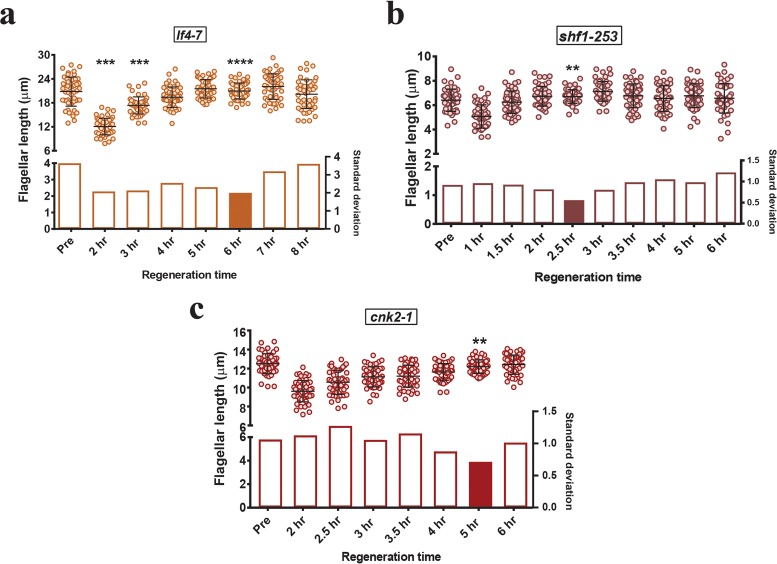
F-L synchronization times differ in mutants of different lengths. For each mutant, distributions of flagellar length during regeneration are shown. (a) Long-flagella mutant *lf4-7*. (b) Short-flagella mutant *shf1-253*. (c) Mild phenotype long-flagella mutant *cnk2-1*. “Pre” represents the steady-state length of the mutant predeflagellation. Bars represent means and standard deviations (top half of each panel). Standard deviations are represented by bar graphs in the lower half of each panel, and the filled bar corresponds to the synchronization time for each mutant on the basis of minimal standard deviation. *n =* 50/each. The *F* test was performed for comparing variance levels with predeflagellation nonsynchronous controls. (a and c) Bonferroni corrected α_altered_ = 0.007; asterisks indicate significant differences below α_altered_ (****, *P* = 0.00005; ***, *P* = 0.0002; **, *P* = 0.004). (b) Bonferroni corrected α_altered_ = 0.0055; asterisks indicate significant differences below α_altered_ (**, *P* = 0.0008).

Flagellar length mutants with both long and short flagella have been previously isolated in *Chlamydomonas* (42–44). Length distributions are reportedly wider in flagellar length mutants than in wild-type cells (43). Therefore, we asked if F-L synchronization would increase our ability to detect length differences in these populations. Some long-flagella mutants have defective regeneration kinetics after amputation (43) and therefore are not suitable for F-L synchronization. However, other mutants have regeneration kinetics comparable to that of wild-type cells (43). We first considered *lf4-7* mutant cells, which have the longest flagella of all identified long-flagella (lf) mutants but can regenerate their flagella with wild-type regeneration kinetics after amputation (44). As expected, prior to deflagellation, the flagellar lengths in the initial population of *lf4-7* cells were distributed very widely (12 µm to 28 µm) (Fig. 3a, upper and lower panels) (Table S4). Following amputation, flagellar length variabilities were reduced at the 2- and 3-h time points, but the mean lengths had not yet achieved the predeflagellation lengths (Fig. 3a). We found that a duration of at least 4 h was required to regenerate flagella to their predeflagellation length. As seen with the wild-type cells, the flagella took extra time after reaching their original length (6 h of regeneration in this case) to become homogeneously distributed (Fig. 3a). Also like wild-type cells, a narrow distribution could be maintained for only a short period of time. For a mutant with short flagella, the *shf1-253* mutant (42), flagella reached their predeflagellation lengths within 1.5 h following deflagellation but took an additional 1 h (2.5 h total) to distribute more narrowly (Fig. 3b, upper and lower panels) (Table S4). Finally, we studied a mutant with only slightly longer flagella than the wild type (45), the *cnk2-1* mutant. Like other flagellar length mutants, these cells regenerated to normal length after 3.5 h following amputation but achieved the narrowest flagellar length distribution at 5 h of regeneration (Fig. 3c, upper and lower panels) (Table S4). These findings suggest that all cells exhibit their tightest flagellar length distribution at a time after they initially reach predeflagellation mean lengths. Therefore, mutants with longer flagella take more time and mutants with shorter flagella take less time to achieve their most narrow flagellar length distribution. Ideally, the optimal time for flagellar synchronization should be adjusted for individual strains.

10.1128/mSphere.00003-17.9 TABLE S4  Flagellar length distribution of length mutants during regeneration. Download TABLE S4, PDF file, 0.1 MB. Copyright © 2017 Dutta and Avasthi. 2017 Dutta and Avasthi This content is distributed under the terms of the Creative Commons Attribution 4.0 International license.

### F-L synchronization may mask important outliers.

**FIG 4:**
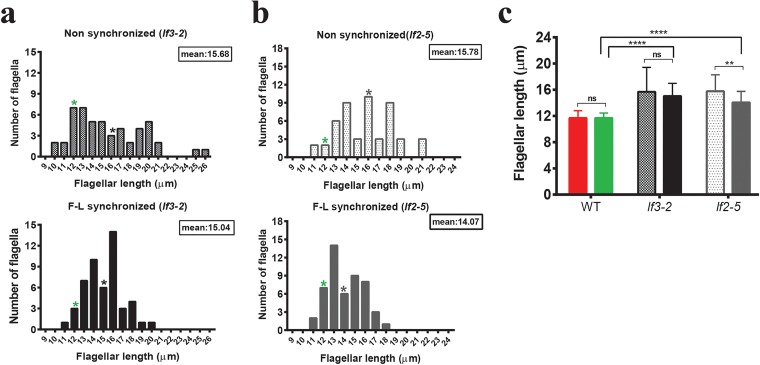
Distributions of flagellar length in long-flagella mutants before and after F-L synchronization. (a) *lf3-2* mutant. (b) *lf2-5* mutant. The upper parts of panels a and b represent the distributions of flagellar lengths in nonsynchronous cells. Cells were then deflagellated by acidic shock and regenerated for 4 h for F-L synchronization. The lower parts of panels a and b correspond to the distributions of flagellar lengths after F-L synchronization. *n =* 50/each (zeros excluded). The mean flagellar length of each mutant is marked by an asterisk. The green asterisks indicate the mean flagellar length observed in wild-type controls. (c) The mean flagellar lengths of wild-type and mutant cells were compared using Bonferroni’s *post hoc* test, and the results are represented. The first and second bars within each pair in panel c represent the mean flagellar lengths of nonsynchronized and synchronized cells, respectively. The Mann-Whitney *U* test was performed for comparing two means in a pair, and Bonferroni’s *post hoc* test was performed for multiple comparisons. Asterisks indicate significant differences (****, *P* ≤ 0.0001; **, *P* ≤ 0.01; ns, *P* > 0.05).

Some flagellar length mutants have a mean flagellar length comparable to that of wild-type cells but have a positively skewed distribution that includes small numbers of mutants with extremely long flagella (43). As F-L synchronization reduces length variance, we asked if the informative long-flagella outliers would be lost after minimizing variability and would thereby decrease our ability to appropriately phenotype this class of mutant. To test this, we chose two long-flagella mutants, mutants *lf2-5* and *lf3-2*, which were able to regenerate their flagella normally and had a large number of flagella in the wild-type range (43). When we induced regeneration for these two mutants for up to 8 h, we found that the F-L synchronization time for both the *lf2-5* mutant and the *lf3-2* mutant was 4 h (Fig. S3). The flagellar length distributions of *lf3-2* cells demonstrated that synchronized cells had a narrow distribution, with flagella no longer than 20 µm and no shorter than 10 µm, which was expected. The synchronized distribution had a negative kurtosis (−0.3738), i.e., a distribution with short tails, compared to the nonsynchronized distribution, which had a positive kurtosis (+0.0358) and relatively long tails. The average length seen with the synchronized population compared to the nonsynchronized population was not changed significantly (Fig. 4a). The mode changed from 12.5 to 16, with the number of cells containing 13 µm to 16 µm long flagella increasing remarkably in the case of F-L synchronized cells (Fig. 4a). Therefore, for the *lf3-2* mutant, F-L synchronization did not affect our ability to identify a mutant phenotype despite eliminating long outliers. In the case of the *lf2-5* mutant, F-L synchronization also removed outliers from the mutant population (Fig. 4b), but this time the average flagellar lengths of nonsynchronized and synchronized cells differed markedly (Fig. 4c) by left-shifting the distribution (Fig. 4b). The mode changed from 16 to 13, and the *lf2-5* mutant showed an average flagellar length value of ~14 µm after F-L synchronization (Fig. 4b), a value which is sometimes seen in wild-type populations (Fig. S4). Also, the nonsynchronized and synchronized kurtosis values (−0.6492 and −0.6753, respectively) were not significantly different. While F-L synchronization of *lf3-2* and *lf2-5* mutants maintained our ability to discriminate between wild-type and mutant phenotypes by reducing the variance (Fig. 4c), the two mutants behave differently with respect to changes in descriptive statistics. Therefore, losing outliers during F-L synchronization has the potential to obscure important information following genetic perturbation. We therefore recommend testing both synchronized and nonsynchronized cells when characterizing new mutants.

10.1128/mSphere.00003-17.3 FIG S3  F-L synchronization time for *lf3-2* and *lf2-5* mutants. For each mutant, distributions of flagellar length during regeneration are shown. (a) *lf3-2* mutant. (b) *lf2-5* mutant. “Pre” represents the steady-state length of the mutant predeflagellation. Bars represent means and standard deviations (top half of each panel). Standard deviations are represented by bar graphs in the lower half of each figure, and the filled bar corresponds to the synchronization time for each mutant on the basis of minimal standard deviation. *n =* 50/each. The *F* test was performed for comparing levels of variance with predeflagellation nonsynchronous controls. For panels a and b, Bonferroni corrected α_altered_ = 0.007 and 0.008, respectively. Asterisks indicate significant differences below α_altered_. (a) ****, *P* = 0.00001; **, *P* = 0.002. (b) **, *P* = 0.004. Download FIG S3, PDF file, 0.1 MB. Copyright © 2017 Dutta and Avasthi. 2017 Dutta and Avasthi This content is distributed under the terms of the Creative Commons Attribution 4.0 International license.

10.1128/mSphere.00003-17.4 FIG S4  Distribution of flagellar lengths in wild-type cells before and after F-L synchronization. Red, nonsynchronized cells; green, synchronized cells. *n =* 50/each. Asterisk, mean flagellar length. Download FIG S4, PDF file, 0.1 MB. Copyright © 2017 Dutta and Avasthi. 2017 Dutta and Avasthi This content is distributed under the terms of the Creative Commons Attribution 4.0 International license.

### Flagellar length variability is related to precursor pool variability.

**FIG 5:**
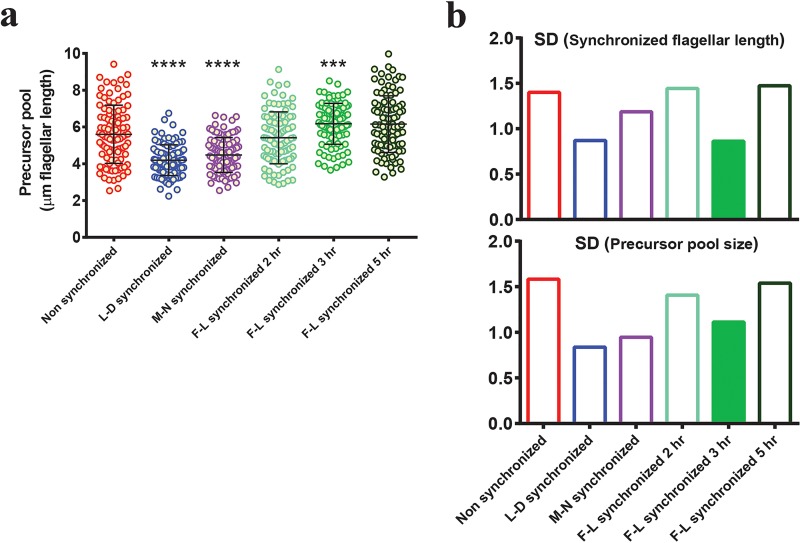
Relationship between flagellar length and precursor pool distribution. (a) Flagellar precursor pool distributions (as determined by regeneration in cycloheximide after the use of different synchronization methods). Red, nonsynchronous; blue, L-D synchronized; purple, M-N synchronized; light green, F-L synchronized for 2 h; medium green, F-L synchronized for 3 h; dark green, F-L synchronized for 5 h. Results of F-L synchronization performed for 3 h show minimal variability among F-L synchronized cells. *n =* 100/each. The *F* test was performed for comparing variance levels (control = nonsynchronous cells). Asterisks indicate significant differences (****, *P* ≤ 0.0001; ***, *P* ≤ 0.001). Bars represent means and standard deviations. (b) Postsynchronization standard deviations (top panel) and corresponding precursor pool standard deviations (bottom panel) of each distribution are represented by bars with matching colors. Cell cycle synchronized cells (L-D synchronized) show the smallest variability in precursor pool distribution (blue, bottom panel). Of the F-L synchronized cells (green), 3 h of synchronization showed the smallest variability in precursor pool distribution (filled bar, bottom panel).

As flagellar synchronization time is highly reproducible within wild-type populations, we hypothesized that there might be an internal regulator which is responsible for the narrow distribution pattern seen after 3 h of regeneration. *Chlamydomonas* cells have a synthesized pool of unassembled flagellar proteins or at least a preexisting pool of some protein that limits the rate of flagellar assembly (termed the precursor pool). The size of this pool is sufficient to assemble flagella to half of their normal length if new protein synthesis is inhibited (46). Limiting-precursor models of flagellar length control have been previously considered, but flagellar length appears to be maintained independently of pool size or concentration (42). However, completely blocking new protein synthesis can limit flagellar length, so we asked if imposing constraints on protein synthesis and incorporation might narrow the resulting flagellar length distribution. In such a case, reduced variability during F-L synchronization would be due to synchronizing flagellar protein synthesis through deflagellation and time-limiting flagellar protein incorporation. In order to test our hypothesis, we determined the variance in the synthesized precursor pool size after the use of different synchronization methods by deflagellating cells and allowing them to regenerate in the presence of cycloheximide (46). This allowed existing flagellar protein to be incorporated into flagella but prevented the synthesis of new protein. In these experiments, flagellar length is a proxy for the amount of limiting protein available for incorporation into flagella without protein synthesis. Precursor pool size is therefore reported in units of micrometers of flagellar length. To evaluate the relationship between flagellar variability and precursor pool variability, we compared flagella that had undergone 3 h of regeneration (tightest distribution) to flagella at two other time points corresponding to increased variability: 2 h and 5 h (Fig. 1b and S2). We performed the same comparison for nonsynchronized, L-D synchronized, and M-N synchronized cells. As expected, the results seen with all cells after synchronization but prior to deflagellation for cycloheximide treatment recapitulated our previous findings (Fig. S5 and Table S5). When we compared precursor pool variance after different regeneration time intervals, we found a narrow distribution of pool sizes at 3 h of regeneration (medium green) but not at 2 h (light green) or at 5 h (dark green) (Fig. 5a and lower half of 5b) (Table S5). Nonsynchronous cells have a precursor pool distribution comparable to that seen at 2 and 5 h of regeneration in F-L synchronized cells (Fig. 5a, red). For all F-L synchronized cells, we observed that the variance seen with the precursor pool (Fig. 5b, lower panel) echoes the postsynchronization length variance (Fig. 5b, upper panel). We also saw that both L-D synchronized cells and M-N synchronized cells showed reduced variance in cytoplasmic precursor pool of flagellar proteins (Fig. 5a, blue and purple, respectively) (Table S5). Because both L-D synchronization and M-N synchronization are cell cycle synchronization methods, we asked next if the narrow precursor pool variability in these cells correlated with a narrow cell size distribution and if we were circumventing the cell size dependence of the flagellar precursor pool during F-L synchronization by time-limiting protein synthesis and incorporation.

10.1128/mSphere.00003-17.5 FIG S5  Predeflagellation flagellar length distribution before precursor pool determination. These data confirm Fig. 1 data showing the narrowest flagellar length distribution for L-D and F-L 3-h synchronized cells. *n =* 100 flagella. Bars represent means and standard deviations. The *F* test was performed for comparing variance levels (control = nonsynchronous cells). Asterisks indicate significant differences (****, *P* ≤ 0.0001; **, *P* ≤ 0.01). Download FIG S5, PDF file, 0.1 MB. Copyright © 2017 Dutta and Avasthi. 2017 Dutta and Avasthi This content is distributed under the terms of the Creative Commons Attribution 4.0 International license.

10.1128/mSphere.00003-17.10 TABLE S5  Flagellar length distribution prior to and after cycloheximide (cyclo) treatment. Download TABLE S5, PDF file, 0.1 MB. Copyright © 2017 Dutta and Avasthi. 2017 Dutta and Avasthi This content is distributed under the terms of the Creative Commons Attribution 4.0 International license.

### Flagellar length synchronization changes the relationship between cell size and precursor pool size.

**FIG 6:**
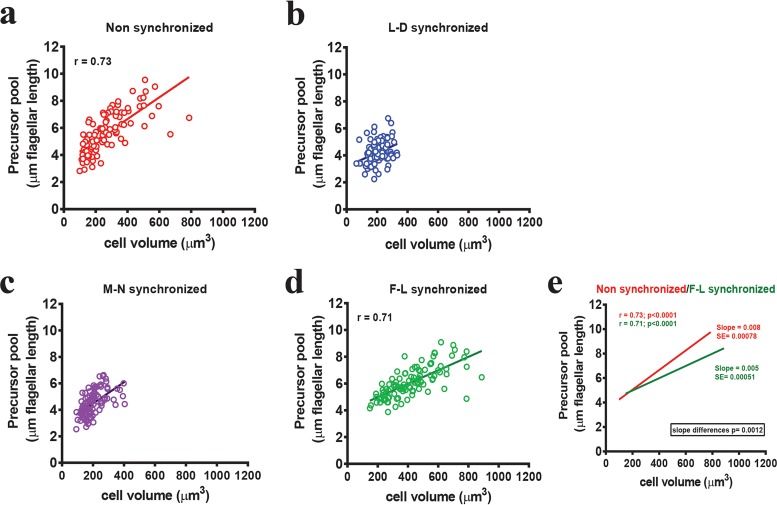
F-L synchronization changes the relationship between the precursor pool size and cell size. Flagellar length in cycloheximide, which corresponds to the precursor pool, is plotted along the *y* axis. Matching cell volume is plotted along the *x* axis. Values are plotted for (a) nonsynchronized (red), (b) L-D synchronized (blue), (c) M-N synchronized (purple), and (d) F-L synchronized (green) populations. *n =* 100. Nonsynchronized cells showed a significant correlation between cell size and precursor pool size (*r* = 0.73). L-D and M-N synchronized cells had narrow cell volume and precursor pool ranges. F-L synchronized cells showed a narrow precursor pool range without limiting cell volume. (e) Relation between nonsynchronous and F-L synchronized cells. The straight lines represent the best-fitted lines through the data point and were drawn after linear regression (red, nonsynchronous cells; green, F-L synchronized cells). SE = standard error of the slopes. The slope became smaller in the case of F-L synchronized cells, a result which is significantly different from that seen with nonsynchronous cells (*P* = 0.0012).

It was previously reported that there is no simple relationship between cell size, flagellar length, and precursor pool size (47). However, we observed that the relative variability of flagellar length across synchronization methods is preserved when considering the variability in pool size (Fig. 5b). Given the general scaling of protein quantity with cell size (48–52), we tested the relationship between cell size and precursor pool size to better understand the factors influencing precursor pool and flagellar length variance across synchronization methods. We regenerated the flagella for 2 h in the presence of cycloheximide after the use of different synchronization methods and measured flagellar length to determine the preexisting precursor pool as before. This time, we also measured the corresponding cell volume. In nonsynchronized cells, cell volumes had a broad distribution (~100 µm^3^ to 900 µm^3^), as did the precursor pool size (~2.5 µm to ~9 µm of flagellar length), with a significant correlation between cell size and precursor pool size (*r* = 0.73, two-tailed *P* < 0.0001) (Fig. 6a). Expectedly, we found that cell volumes were very restricted, ranging from ~100 µm^3^ to ~400 µm^3^, in both L-D synchronized cells and M-N synchronized cells (Fig. 6b and c, respectively), as they are cell cycle synchronized. Since smaller cells generally produce less protein (48), the restricted cell volumes of cell cycle synchronized populations also limited the protein precursor pool size to within a very narrow range (~4.5 µm of flagellar length) (Fig. 6b and c). On the other hand, like the nonsynchronous cells, F-L synchronized cells had a large cell size range (~100 µm^3^ to 900 µm^3^). However, unlike nonsynchronous cells, which had a precursor pool size range of ~6.5 µm of flagellar length (Fig. 6a), F-L synchronized cells had a narrow precursor pool range (~5 µm flagellar length) more comparable to the precursor pool size in cell cycle synchronized cells (Fig. 6d). While a correlation between cell size and precursor pool size was still maintained in F-L synchronized cells (*r* = 0.71, two-tailed *P* < 0.0001), the slopes of the regression lines for nonsynchronous cells and F-L synchronized cells were significantly different (*P* = 0.0012) (53–55) (Fig. 6e, red line and green line, respectively). In other words, the relationship between cell size and available precursor pool was altered upon F-L synchronization. Presumably, F-L synchronization can limit the precursor pool sizes by limiting the amount of time during which the precursor pool can accumulate without the need to limit pool size by restricting cell size through cell cycle synchronization.

## DISCUSSION

Here we have shown a powerful new approach to improve understanding of ciliary length-related biology by characterizing a synchronization method that minimizes flagellar length variability. It is well established that flagellar length variability can be controlled by restricting the cell size (the basis of cell cycle synchronization). Our data suggest that the size of the precursor pool (the existing pool of flagellar protein not assembled into flagella) is also related to the cell size. Limiting flagellar protein is not currently considered a major factor controlling flagellar length because the amount of flagellar proteins in *Chlamydomonas* clearly exceeds the amount assembled into flagella, the size of the pool of unassembled flagellar precursors does not correlate with flagellar length during assembly, and flagellar length does not appear to be strongly dependent upon the number of flagella in mutants with variable flagellar numbers (41, 47, 56). However, while the precursor pool size is correlated with cell size, cell cycle synchronization methods severely limit both cell size and precursor pool variability. We further found that we can circumvent cell size restrictions for minimizing flagellar length variability by limiting the amount of time that the flagellar precursor pool can accumulate and incorporate into flagella. It is well known that mammalian cell ciliary studies often initiate ciliogenesis by serum starvation of confluent cells (5, 19). By standardizing plating density and limiting serum starvation time prior to subsequent experimentation (thereby limiting the time window of assembly), the F-L synchronization method may be applicable to studies in mammalian cells.

**FIG 7:**
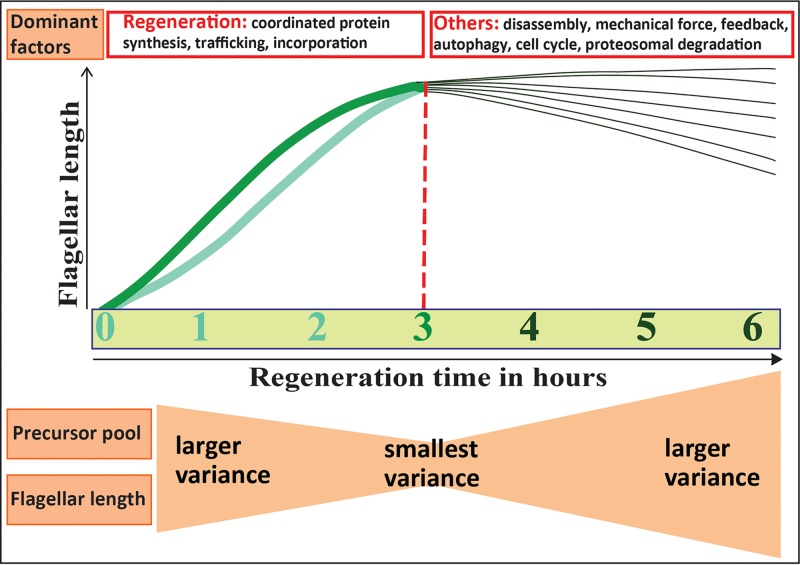
Proposed model of flagellar length synchronization. Thick light green and medium green lines represent slow-growing and fast-growing flagella, respectively. The red dotted line denotes the 3 h postdeflagellation time at which flagellar length variability was minimized. Regeneration is initially the dominant factor. With time, other listed factors may contribute, resulting in higher levels of flagellar length variability.

Assembly of full-length flagella requires a preexisting precursor pool, *de novo* synthesis of flagellar precursor proteins, and also incorporation of those proteins into the flagellar structure (57). The expression of genes encoding ciliary proteins is dramatically upregulated after flagellar amputation to replenish the precursor pool and to provide the proteins required for flagellar assembly (58). We propose a model for F-L synchronization (Fig. 7) where F-L synchronization via deflagellation works by stimulating a highly regulated program of gene expression and flagellar protein incorporation so that all cells can regenerate their flagella synchronously regardless of their divisional phase. Synchronization is then achieved by limiting the time window (Fig. 7, red dotted line) during which the cells are allowed to regenerate their flagella (3 h). Combining a simultaneous induction of the regeneration program and a restriction of the amount of protein synthesis and incorporation results in a tighter length distribution pattern.

If time limiting protein synthesis and incorporation results in a narrow distribution of flagellar lengths, why was there increased variability of flagellar lengths at an earlier flagellar synchronization interval (2 h)? Rates of flagellar regeneration differ from cell to cell; some flagella are fast growing (Fig. 7, medium green line) and attain their original length within 2 h of amputation, while slow-growing flagella (Fig. 7, light green line) can reach only 80% of their length within that period. We saw in measurements of unassembled flagellar protein (Fig. 5b, lower panel) (see Table S5 in the supplemental material) that cells that have undergone 2 h of F-L synchronization have a smaller precursor pool (mean, 5.4 µm of flagellar length) than those that have undergone 3 h of synchronization (mean, 6.2 µm of flagellar length). The slow growth of some cells at 2 h postdeflagellation may therefore be due to reduced protein synthesis and accumulation. We propose that, as we extended the regeneration time beyond 2 h, slow-growing flagella finally reached their original length and fast-growing flagella approached their maximum length by reducing their assembly rate and reaching equilibrium with continuous disassembly (Fig. 7). To confirm this, we would need data at the individual-cell level rather than at the population level, which will be obtained in future studies by trapping individual motile cells in a microfluidic chamber (59).

While all cells must initiate a regeneration program upon deflagellation, with increasing time, the deflagellation-induced protein synthesis and incorporation program (which decreases as a function of time and flagellar length) may be overcome by other regulating factors such as disassembly, mechanical force, proteosomal degradation, feedback control, and autophagy (60–64) (Fig. 7, dark green lines). Also, when the regeneration program no longer drives flagellar length after 3 h, cell cycle regulation may dominate, resulting in the heterogeneous flagellar length and precursor pool size distributions seen in nonsynchronized cells.

In addition to maximizing our ability to detect effects in inhibitor studies, we observed that F-L synchronization can be readily applied to genetically perturbed length mutants to reduce their length heterogeneity. All mutants have different genetic defects, and we showed that several mutants responded differently to synchronization, highlighting that both synchronized cells and nonsynchronized cells should be tested when phenotyping newly identified mutants. Interestingly, we were able to discriminate long-flagella mutants from wild-type cells on the basis of the synchronization time alone, regardless of mean length. In other words, when F-L synchronization eliminated important outliers and reduced the ability to discriminate on the basis of mean flagellar length, cells still showed a flagellar synchronization profile more similar to that of long-flagella mutants (4 to 5 h synchronization time) than to that of wild-type cells (3 h synchronization time). This suggests that flagellar synchronization time itself can be a useful phenotyping parameter.

Currently, the most commonly used method of reducing flagellar length variability in *Chlamydomonas* is cell cycle synchronization using L-D cycling. However, in L-D synchronized cells, natural variance in cells (8, 35) prevent 100% synchronization of flagellar length. Using F-L synchronization, we can synchronize 100% of the population through deflagellation and produce a homogeneous distribution of length by 3 h. Conventional L-D synchronization, in contrast, requires at least 3 days to achieve comparable levels of homogeneity. F-L synchronization does not require a dark chamber with automated light switching. Moreover, the entire experiment can be performed in a rich medium such as Tris-acetate-phosphate (TAP) medium instead of minimal medium, which is very sensitive to changes in pH. This facilitates the use of inhibitors that would otherwise dramatically affect the pH of the medium. F-L synchronization showed effects of length-altering chemicals on flagellar length that were equivalent to or stronger than those seen with L-D synchronization, demonstrating its utility in addition to its uniformity and simplicity.

The results presented here facilitate identification of ciliary length-related defects (65–68) by increasing our ability to detect small changes in ciliary size but, more broadly, help us understand factors affecting ciliary length regulation. By inducing a fully synchronous cellular program (regeneration) that temporarily dominates multiple other factors to minimize flagellar heterogeneity, F-L synchronization also has the strong potential to benefit studies of ciliary motility or ciliary signaling.

## MATERIALS AND METHODS

### Strains and length-altering chemical treatments.

*Chlamydomonas reinhardtii* wild-type 137c mt+ (CC125), *lf4-7* mt− (CC4534), *shf1-253* mt− (CC2348), *cnk2-1* (CC4689), *lf2-5* mt− (CC2287), and *lf3-2* mt− (CC2289) strains were obtained from the *Chlamydomonas* Resource Center at the University of Minnesota. All chemicals were purchased from Sigma (St. Louis, MO), and final concentrations of 0.4 mM IBMX, 10 mM NaPPi, 10 µM LatB, 25 mM LiCl, and 10 µg·ml^−1^ cycloheximide were used. Compounds were diluted to the indicated doses either with TAP medium or with 100% dimethyl sulfoxide (DMSO). For the chemical treatment, 1 or 2 ml of cells was treated with the indicated concentration of chemicals with the indicated controls and placed on a rotator for 90 min or 120 minutes as indicated in the text.

### Culture condition and different synchronization methods.

All cells were maintained on TAP plates containing 1.5% agar (Difco Laboratories, Detroit, MI) (24). For liquid cultures, cells were inoculated from TAP plates at less than 2 weeks of age.

### Nonsynchronous culture.

For nonsynchronous culture, cells were grown in liquid TAP medium for 24 h on a culture rotator drum at 25°C under conditions of continuous illumination with a light-emitting diode (LED) LumiBar with independent red and blue light control (LumiGrow, Inc.).

### Light/dark (L-D) synchronization.

Cells were inoculated in minimal medium (M1 medium) from the TAP plates and kept in light for 12 h and then in dark for 12 h, alternating at 25°C for at least 3 days. After each L/D cycle (12 h/12 h), cultures were diluted to 2 × 10^5^ cells·ml^−1^ with fresh M1 medium. On the fourth day, after growing in the light phase for 5 h, cultures were immediately transferred to TAP medium prior to chemical treatment.

### Synchronization by nitrogen starvation (M-N synchronization).

M-N synchronization was attained by inducing gametogenesis in nitrogen-free minimal medium for 18 to 20 h in continuous light at 25°C under a LumiBar. These cells were then transferred to TAP medium for 4 h prior to chemical treatment.

### Flagellar length synchronization (F-L synchronization).

For F-L synchronization, *Chlamydomonas* cells were grown in liquid TAP medium and then induced to regenerate flagella after acid-mediated flagellar excision (69). Acetic acid (60 µl of 0.5 N) was added to 1 ml of cells for deflagellation (pH = 4.5). Immediately after 45 s, 70 µl of 0.5 N KOH was added to neutralize the medium, which ultimately induced the flagellar regeneration. Wild-type cells were grown for 3 h for flagellar regeneration under conditions of continuous illumination with a LumiBar on a rotator drum.

### Flagellar length and cell volume measurement.

For measurements of flagella, cells were fixed in 1% glutaraldehyde and kept at 4°C. Cells were then centrifuged at 1,000 × *g* for 1 min and mounted between a glass slide and coverslip. Imaging was performed using a Zeiss Axioscope differential interference contrast (DIC) microscope with a 40× objective lens and a Zeiss AxioCam 105 color camera. Flagellar length measurements were done by analysis of line segments and spline fitting using ImageJ software (NIH, USA). All flagella in a particular field were considered, and at least 50 flagella were measured at each time point. For cell size determination, cell volumes were calculated using the ellipsoid equation 4/3π (*L*/2)(*W*/2)^2^, where *L* is cell length and *W* is cell width (70). Flagellar length distributions and cell volumes were plotted using GraphPad Prism software version 6 (GraphPad, USA).

### Flagellar precursor pool determination.

For flagellar precursor pool determination, cells were allowed to regenerate their flagella in the presence of 10 µg·ml^−1^ cycloheximide following deflagellation (46). Cells processed using different synchronization methods were induced to regenerate flagella after acidic shock and were then returned to neutral pH by addition of KOH as described above and subjected to cycloheximide treatment immediately. For precursor pool determination in F-L synchronized cells, cells were allowed to regenerate their flagella for 2 h, 3 h, and 5 h after the first deflagellation and then subjected to a second deflagellation prior to cycloheximide treatment. For all cases, cells were centrifuged at 1,000 × *g* for 2 min after neutralization and were then resuspended in TAP medium containing cycloheximide. Cells were placed on a rotator for 120 min, and flagellar length measurements were carried out to determine the amount of unassembled limiting flagellar protein.

### Statistical analysis.

Statistical analyses were performed using GraphPad Prism software version 6 and Microsoft Excel-2010. Descriptive statistics were expressed as means and standard deviations. *F* tests were performed in Excel to compare the levels of variance of the data in the data set and to determine the *P* values (by two-tailed test). The nonparametric Mann-Whitney *U* test was performed for comparing two means. One-way analysis of variance (ANOVA) and Bonferroni’s *post hoc* tests were performed for multiple comparisons and to determine *P* values. For all data sets, *P* values of <0.05 were considered statistically significant. However, Bonferroni’s correction was applied when multiple pairwise comparisons were performed on a single set of data and *P* values were adjusted accordingly. Frequency distributions and column statistics were used to determine mode and kurtosis, respectively. For determining Pearson *r* values, we performed correlation analyses in GraphPad Prism. Slopes and associated standard errors (SE) were determined using linear regression (least-squares fit), also in GraphPad Prism. We also determined the difference between the two slopes in Fig. 6e using an available online calculator (53, 54).
