# Supplementary figures and images for: Flagellar Synchronization Is a Simple Alternative to Cell Cycle Synchronization for Ciliary and Flagellar Studies
Source: mSphere. 2017 Mar 8;2(2):e00003-17. doi: 10.1128/mSphere.00003-17 (PMC5343170; doi:10.1128/mSphere.00003-17)

**Figure S1:**

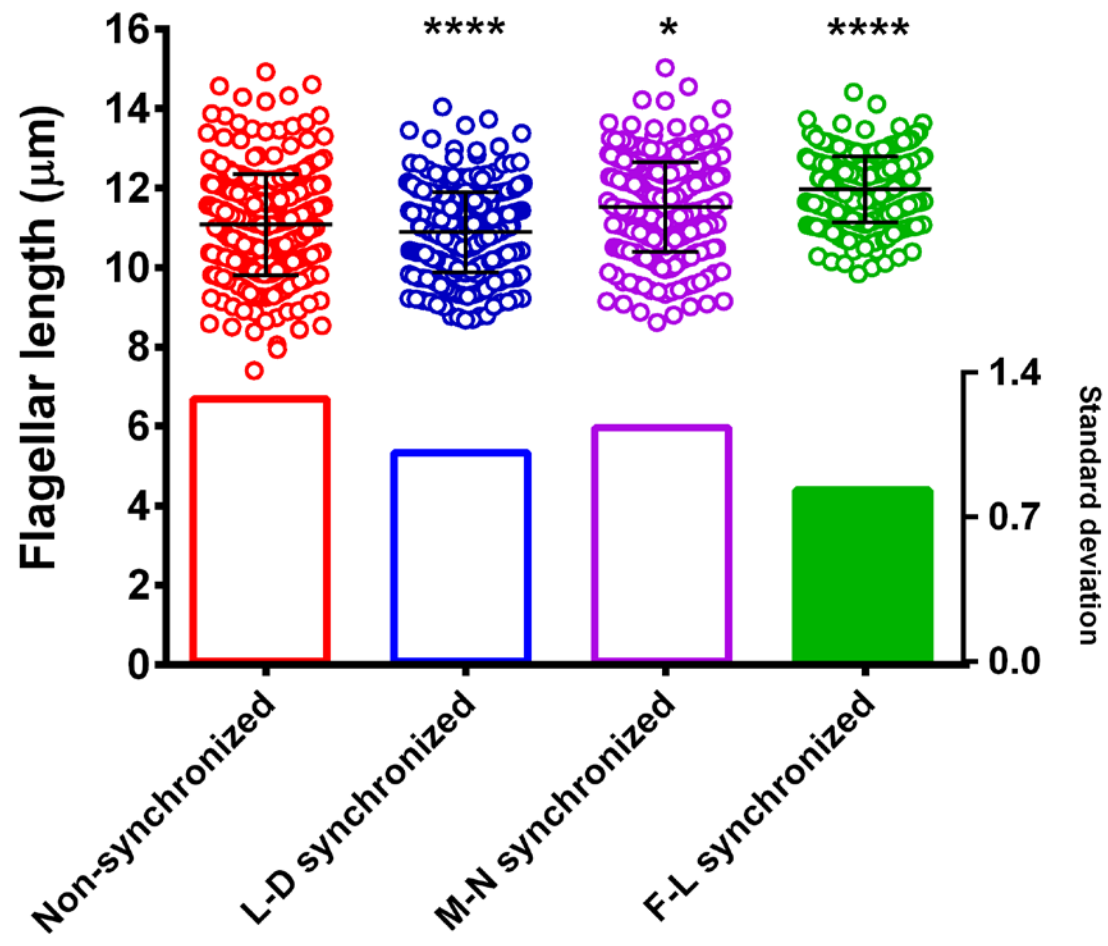

Supplement: FIG S1 [file sph002172246sf1.pdf]

**Figure S2:**

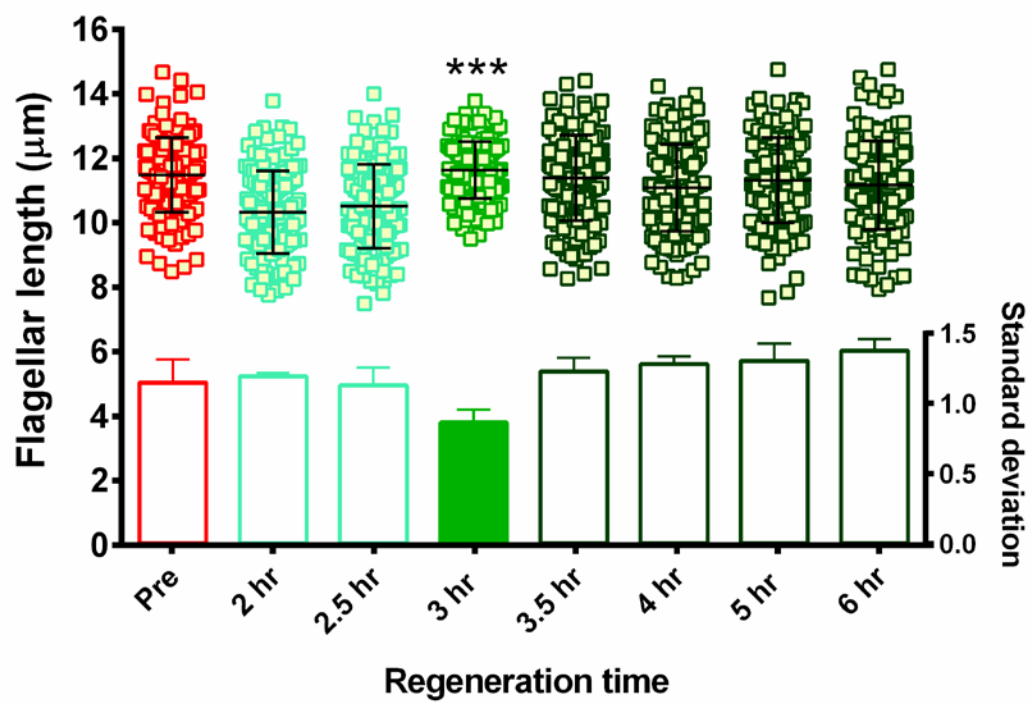

Supplement: FIG S2 [file sph002172246sf2.pdf]

**Figure S3:**

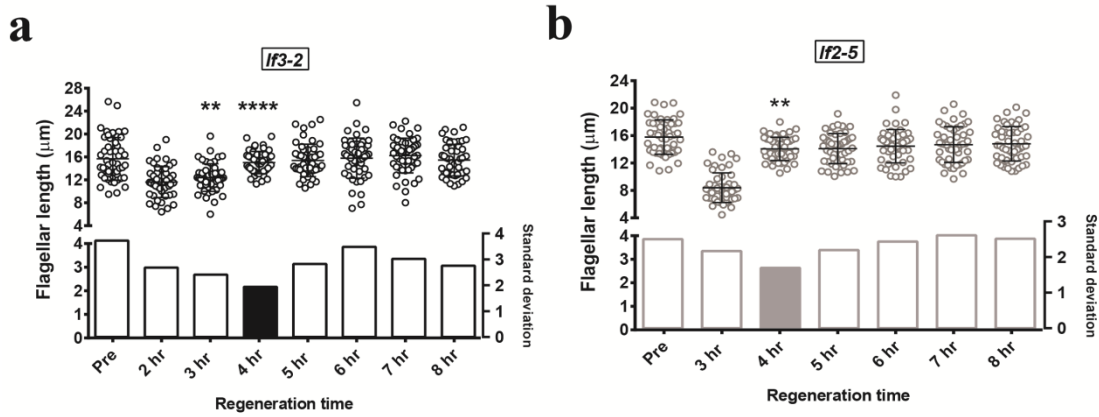

Supplement: FIG S3 [file sph002172246sf3.pdf]

**Figure S4:**

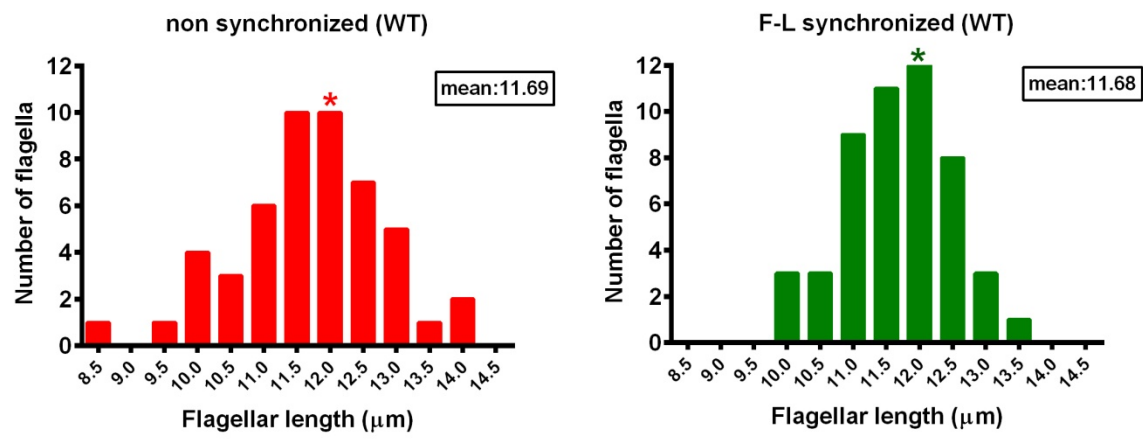

Supplement: FIG S4 [file sph002172246sf4.pdf]

**Figure S5:**

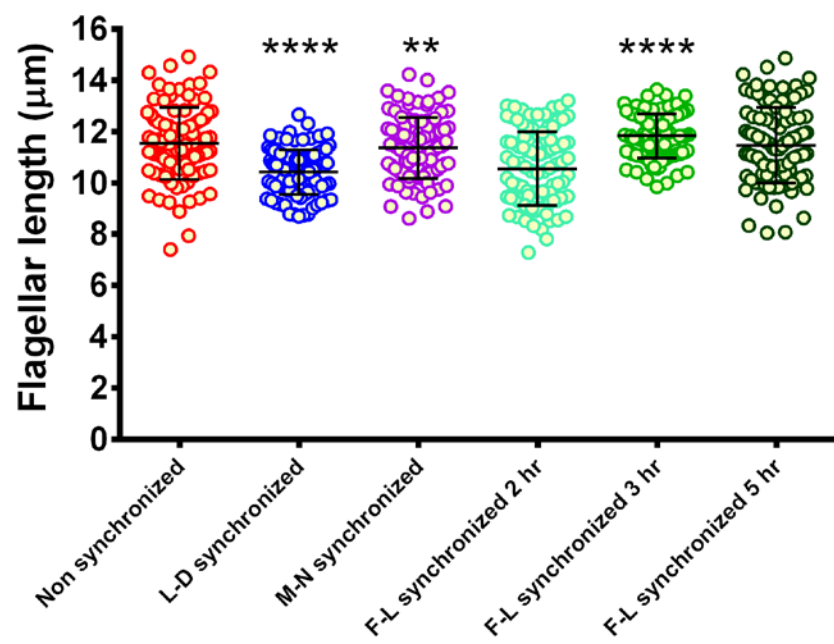

Supplement: FIG S5 [file sph002172246sf5.pdf]
