# Supplementary material for: Flagellar Synchronization Is a Simple Alternative to Cell Cycle Synchronization for Ciliary and Flagellar Studies
Source: mSphere. 2017 Mar 8;2(2):e00003-17. doi: 10.1128/mSphere.00003-17 (PMC5343170; doi:10.1128/mSphere.00003-17)
Supplement: TABLE S1 [file sph002172246st6.pdf]

Table S1

	Non-synchronized	L-D synchronized	M-N synchronized	F-L synchronized
**Mean**	11.54	11.35	12.11	11.83
**SD**	1.401	0.9614	1.059	0.8633
