# Supplementary material for: Flagellar Synchronization Is a Simple Alternative to Cell Cycle Synchronization for Ciliary and Flagellar Studies
Source: mSphere. 2017 Mar 8;2(2):e00003-17. doi: 10.1128/mSphere.00003-17 (PMC5343170; doi:10.1128/mSphere.00003-17)
Supplement: TABLE S2 [file sph002172246st7.pdf]

Table S2

	Pre	2 hour	2.5 hour	3 hour	3.5 hour	4 hour	5 hour	6 hour
**Mean**	11.70	11.00	11.40	11.68	12.12	11.64	11.54	11.26
**SD**	1.117	1.221	.9902	.7587	1.117	1.225	1.324	1.472
