# Supplementary material for: Flagellar Synchronization Is a Simple Alternative to Cell Cycle Synchronization for Ciliary and Flagellar Studies
Source: mSphere. 2017 Mar 8;2(2):e00003-17. doi: 10.1128/mSphere.00003-17 (PMC5343170; doi:10.1128/mSphere.00003-17)
Supplement: TABLE S3 [file sph002172246st8.pdf]

Table S3

**Chem-ical**		**Non-synchronized**		**L-D synchronized**		**M-N synchronized**		**F-L synchronized**	
		Mean	SD	Mean	SD	Mean	SD	Mean	SD
**IBMX**	Control	11.03	1.225	10.65	1.029	11.35	0.9315	10.70	1.029
	Treated	7.063	1.103	5.965	1.122	6.657	1.015	5.863	1.015
**LatB**	Control	12.11	1.052	10.65	1.029	11.31	1.200	11.63	0.8298
	Treated	9.122	1.337	7.965	1.164	9.079	1.155	8.407	0.8109
**NaPPi**	Control	11.07	1.195	10.42	0.8714	10.89	0.8654	11.94	0.8855
	Treated	7.54	1.165	6.198	1.141	7.244	1.324	6.449	0.8925
**LiCl**	Control	11.33	1.118	10.42	0.8714	12.10	1.051	12.13	0.8005
	Treated	14.49	2.342	14.68	1.479	15.53	2.487	16.94	1.468
