# Supplementary material for: Flagellar Synchronization Is a Simple Alternative to Cell Cycle Synchronization for Ciliary and Flagellar Studies
Source: mSphere. 2017 Mar 8;2(2):e00003-17. doi: 10.1128/mSphere.00003-17 (PMC5343170; doi:10.1128/mSphere.00003-17)
Supplement: TABLE S4 [file sph002172246st9.pdf]

Table S4

	**Time After Regeneration**										
lf4-7		**Pre**	**2 hr**	**3 hr**	**4hr**	**5 hr**	**6hr**	**7 hr**	**8 hr**		
	Mean	20.8	12.07	17.36	19.42	21.51	**20.96**	22.07	20.16		
	SD	3.64	2.08	2.13	2.55	2.31	**1.99**	3.19	3.61		
shf1-253		**Pre**	**1 hr**	**1.5 hr**	**2hr**	**2.5 hr**	**3hr**	**3.5 hr**	**4 hr**	**5 hr**	**6 hr**
	Mean	6.4	5.07	6.24	6.73	**6.7**	7.13	6.78	6.54	6.77	6.56
	SD	0.92	0.96	0.92	0.81	**0.56**	0.80	0.98	1.05	0.98	1.21
cnk2-1		**Pre**	**2 hr**	**2.5 hr**	**3hr**	**3.5 hr**	**4 hr**	**5 hr**	**6 hr**		
	Mean	12.5	9.61	10.55	11.12	11.20	11.64	**12.2**	12.41		
	SD	1.05	1.12	1.27	1.05	1.15	0.87	**0.71**	1.01		
