# Supplementary material for: Flagellar Synchronization Is a Simple Alternative to Cell Cycle Synchronization for Ciliary and Flagellar Studies
Source: mSphere. 2017 Mar 8;2(2):e00003-17. doi: 10.1128/mSphere.00003-17 (PMC5343170; doi:10.1128/mSphere.00003-17)
Supplement: TABLE S5 [file sph002172246st10.pdf]

Table S5

		Non synchronized	L-D synchronized	M-N synchronized	F-L synchronized		
					**2 hr**	**3 hr**	**5 hr**
**Before cyclo**	Mean	11.54	10.42	11.36	10.56	11.83	11.46
	SD	1.401	0.871	1.188	1.444	0.863	1.474
**After cyclo**	Mean	5.600	4.191	4.513	5.409	6.162	6.156
	SD	1.583	0.837	0.917	1.408	1.097	1.54
